# Response Properties of Motor Equivalence Neurons of the Primate Premotor Cortex

**DOI:** 10.3389/fnbeh.2017.00061

**Published:** 2017-04-12

**Authors:** Eleftherios Neromyliotis, A. K. Moschovakis

**Affiliations:** ^1^Institute of Applied and Computational Mathematics, Foundation for Research and TechnologyHeraklion, Greece; ^2^Department of Basic Sciences, Faculty of Medicine, University of CreteHeraklion, Greece

**Keywords:** saccades, hand movements, arcuate sulcus, eye-hand coordination, establishment of synergies

## Abstract

To study the response properties of cells that could participate in eye-hand coordination we trained two macaque monkeys to perform center-out saccades and pointing movements with their right or left forelimb toward visual targets presented on a video display. We analyzed the phasic movement related discharges of neurons of the periarcuate cortex that fire before and during saccades and movements of the hand whether accompanied by movements of the other effector or not. Because such cells could encode an abstract form of the desired displacement vector without regard to the effector that would execute the movement we refer to such cells as motor equivalence neurons (Meq). Most of them (75%) were found in or near the smooth pursuit region and the grasp related region in the caudal bank of the arcuate sulcus. The onset of their phasic discharges preceded saccades by about 70 ms and hand movements by about 150 ms and was often correlated to both the onset of saccades and the onset of hand movements. The on-direction of Meq cells was uniformly distributed without preference for ipsiversive or contraversive movements. In about half of the Meq cells the preferred direction for saccades was the preferred direction for hand movements as well. In the remaining cells the difference was considerable (>90 deg), and the on-direction for eye-hand movements resembled that for isolated saccades in some cells and for isolated hand movements in others. A three layer neural network model that used Meq cells as its input layer showed that the combination of effector invariant discharges with non-invariant discharges could help reduce the number of decoding errors when the network attempts to compute the correct movement metrics of the right effector.

## Introduction

Most of the things we do in everyday life depend on the execution of well-orchestrated sequences of coordinated movements of the hands and the eyes (Land and Hayhoe, 2001). Several parameters of the movements of one of the effectors such as the size of the gaze shifts, and the target and duration of fixations are severely constrained by the movements of the other (Land et al., 1999; Johansson et al., 2001). Typically, eye movements are tightly coupled, in time and space, to the hand movements they generally precede by tens of milliseconds. Coordination of the eyes and the hand with an accuracy of a few centimeters and milliseconds seems to be needed for the adequate performance of some of these tasks (Land and McLeod, 2000). Given such routine observations, it is reasonable to ask if eye and hand responses are initiated by one common command signal or by different command signals (Bekkering et al., 1994). A correlational approach has often been adopted in efforts to address this question. To this end, the relationship between the reaction times of the eyes and the hand has been the object of considerable study with the underlying assumption that the lower the correlation the likelier the independence of the commands driving eyes and hand. These have been found to hover at around 0.5 (Prablanc et al., 1979b; Gielen et al., 1984; Fischer and Rogal, 1986) but higher or lower values have also been reported leading different authors to different conclusions. Exploration of possible spatial coupling between ocular and manual responses has had a similarly inconclusive outcome. For example, Nemire and Bridgeman (1987) concluded “that the two systems share a single map of space” after asking subjects to execute rapid eye movements and point a hand held pointer to the location of a target. In their experiments, both saccades and pointing were affected in a similar way and saccadic and manual measures were highly correlated when the oculomotor system was interfered with. On the other hand, no correlation between the variable errors of saccades and hand movements were found in step, gap, memory, scanning and antisaccade/antireach tasks (Sailer et al., 2000).

To determine if one signal or two different signals command the eye and hand components of eye-hand movements it is meaningful to study the responses of neurons whose discharge accompanies both eye and hand movements. The premotor cortex (Brodman's area 6) is eminently deserving of such a search. When one signs one's name with the index finger or the toe, the region of the primary motor cortex activated depends on the effector performing the movement whereas one region of the premotor cortex is activated regardless of the effector used (Rijntjes et al., 1999). Also, the premotor cortex contains cells discharging when either the ipsilateral or the contralateral limb moves toward a target (Hoshi and Tanji, 2002) and cells that discharge when either a saccade or a hand movement is executed (Fujii et al., 2000).

The present report is part of a long-term effort to understand how the premotor (PM) cortex contributes to the coordination of eye, head and hand movements. Earlier work from our laboratory demonstrated that the posterior bank of the arcuate sulcus (AS) of monkeys contains cells oligosynaptically connected to the lateral rectus muscle and is activated for saccades (Moschovakis et al., 2004), a finding that was later verified by us (Savaki et al., 2015) and others (Koyama et al., 2004; Baker et al., 2006). The same region has been traditionally associated with the control of grasping movements (Matelli et al., 1991). It projects to the “hand” area of the primary motor cortex (He et al., 1993) as well as upper cervical segments (Martino and Strick, 1987; He et al., 1993; Dum and Strick, 2002) usually associated with the control of neck muscles and proximal/axial body musculature. Some of its neurons discharge for grasping movements (Raos et al., 2006) and others for reaching movements (Godschalk et al., 1981) while its electrical stimulation evokes proximal and distal forelimb movements (Godschalk et al., 1995; Dum and Strick, 2002). Here, we demonstrate that it also contains cells that emit phasic discharges for both saccades and hand movements and provide a detailed description of their response properties.

## Methods

### Animal preparation

We obtained data from three hemispheres of two adult female rhesus monkeys (*macaca mulatta*), weighing 5.2 and 6 kg, respectively. They were purpose-bred by authorized suppliers within the European Union (Deutsches Primatenzentrum, Gottingen, Germany). Experimental protocols were approved by the Institutional Ethics Committee of FORTH and the Veterinary authorities of the Region of Crete and complied with European (directive 2010/63/EU and its amendments) and National (Presidential Decree 56/2013) laws on the protection of animals used for scientific purposes. Subjects were surgically prepared for painless head immobilization, eye position monitoring and extracellular recording under anesthesia and aseptic conditions. For head immobilization, a metal bolt was cemented onto mandibular plates secured on the cranium with titanium screws (Synthes, Bettlach, Switzerland). After training was completed and following craniotomy, a metal chamber (Crist Instr., Damascus, MD) of 1 cm in radius was cemented onto the bone. Recording chambers were centered at stereotaxic coordinates 21 mm anterior to the interaural line and 16 mm lateral to the midsagittal plane, first on the left and then on the right side, of subject R and 16 mm anterior to the interaural line and 16 mm lateral to the midsagittal plane on the left side of subject L. In between recording sessions, the chamber was filled with a gel containing antibiotic (Tobramycin 0.3%) and capped. To monitor eye movements (Robinson, 1963), a scleral search coil (AS633 Cooner wire, Chatsworth, CA) was sutured on the sclera (modified from Judge et al., 1980).

### Behavioral paradigm

Monkeys were trained for 3–6 months till they successfully completed more than 90% of the trials. To perform their tasks, subjects sat in a primate chair in the dark, facing a 21″ 120 Hz monitor (MicroTouch 3M, St. Paul, MN), positioned 27 cm in front of their head, which was centered within two orthogonal magnetic fields generated by currents alternating at 50 and 75 kHz, respectively. The current induced in the eye coil was demodulated (Remmel labs, Ashland, Ma) to obtain the vertical and horizontal components of instantaneous eye position (Remmel, 1984). System gain was calibrated frequently, by averaging at least 10 movements in each direction after asking the animal to execute a series of vertical and horizontal movements of 10° amplitude centered on straight ahead.

White and circular targets (1° in diameter) were presented on the video monitor. A liquid delivery tube was attached close to their mouth, and successful completion of each trial was rewarded with apple juice. Hand tasks were performed with the forelimb contralateral to the hemisphere we recorded from. As in previous studies (e.g., Boucher et al., 2007), to move a cursor on the screen (a white open circle of 1°) with their hands, subjects handled a joystick (ETI systems, Carlsbad, CA) that was placed at hip level and could not be seen by the subject. Eye position and joystick output were sampled at rates of 1,000 and 666 Hz, respectively, with an A/D converter (Cambridge Electronics Design - CED - micro1401-3, Cambridge, UK) of a microcomputer running the Spike2 software (CED, Cambridge, UK), and stored on disk for off-line analysis.

**Figure 1:**
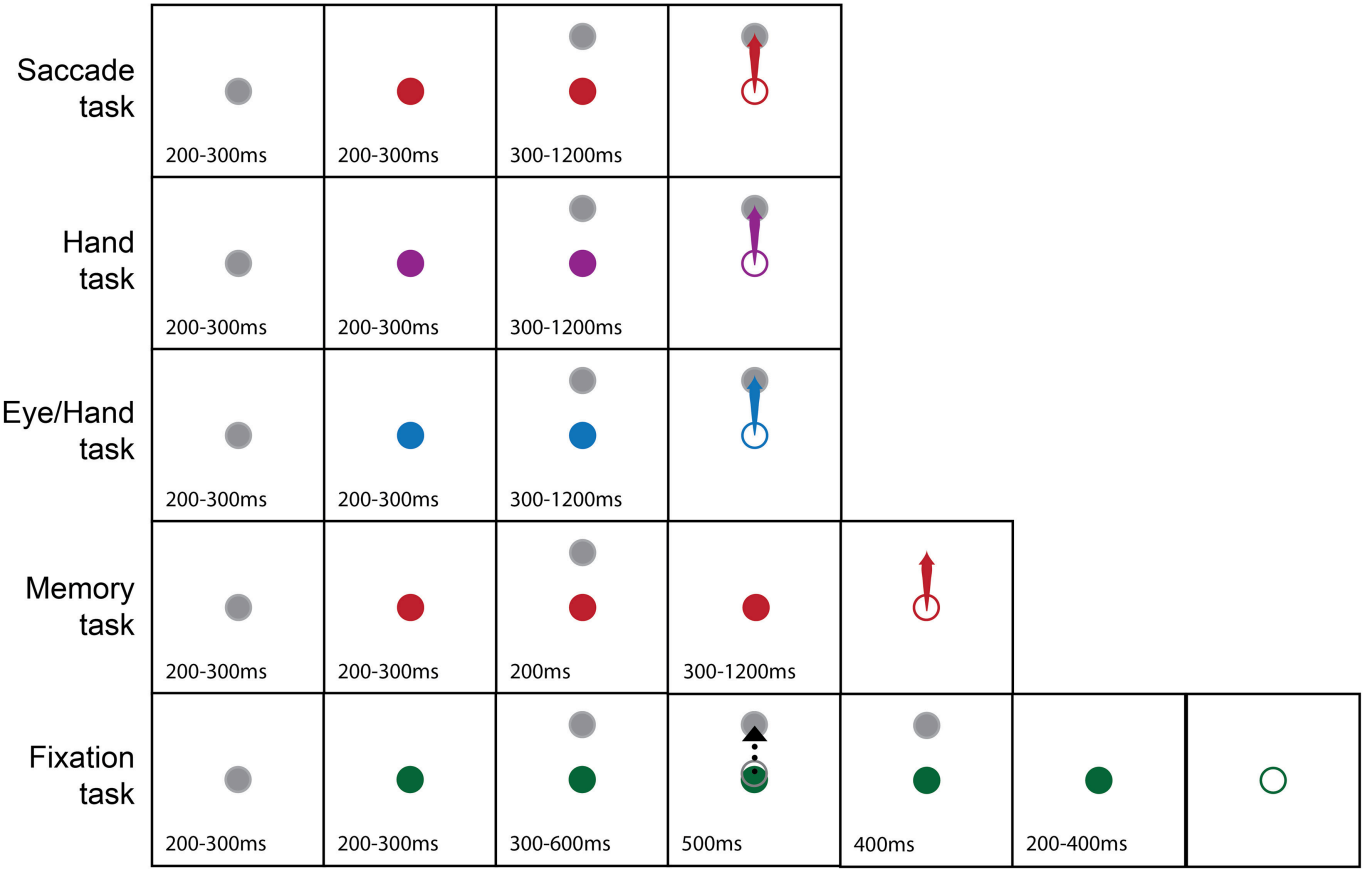
**Pictorial summary of the behavioral tasks we used**. The gray fixation point turned into a color (solid circle) to instruct the subject on the effector to use (Red, Eye; Purple, Hand; Blue, Both the eyes and the Hand; Green, Hold fixation) to capture a peripheral target (arrow). The dashed black arrow indicates cursor movement in the fixation task. Open circles indicate the appearance of the Go cue. Numbers indicate epoch duration (in ms).

Subjects performed center-out tasks using their eyes, their hand or both. Figure 1 illustrates the tasks we employed. Each trial started with the appearance of a white fixation disk in the center of the screen, which, after 200–300 ms changed color depending on the effector (instruction cue) that had to be used in the successful completion of the trial (red for saccades, purple for hand movements, blue for coordinated eye-hand movements). After another 200–300 ms, a peripheral target (1° diameter) appeared in one of 8 different directions (0° = right, 45° = right-up, 90° = up, 135° = up-left, 180° = left, 225° = down-left, 270° = down, 315° = down-right) and at a distance of 10° or 20° away from the fovea. After a variable delay (300–1,200 ms), the fixation point turned into an open circle (the go cue) of the same diameter and color as the fixation stimulus. Subjects had to keep their eyes as well as the hand-controlled cursor within a square window, 1.5° on the side, surrounding the fixation spot for the whole duration of the trial up until the appearance of the Go cue. Following this, subjects had to execute a movement toward the target using the effector(s) prescribed by the color of the instruction cue. In the case of hand movements, subjects were required to move the aforementioned white open circle cursor on the screen with the help of the handled joystick. When a single effector hand movement was performed, the line of sight had to remain fixated in the center of the screen. In turn, in single effector saccade trials, the hand controlled cursor had to remain in the center of the screen. After acquiring the target, the animal had to keep the effector(s) within a square window 1.5° on the side surrounding the target's center for 200 ms, for the trial to be considered successful whereupon the subject was rewarded with a drop of liquid delivered through a computer controlled valve (Crist instruments, Damascus, MD). In cases when the animal moved the wrong effector, or the movement metrics were wrong (i.e., the effector landed outside the square window surrounding the target's center) or the movement was not completed within 0.6 s (for eye movements) or 1.2 s (for hand movements), the trial was aborted and there was no reward. In another task, the subject had to execute a rapid eye movement to the memorized location of a flashed target. This task was similar to the visually guided saccade task in all aspects except that the peripheral target was shown for 200 ms. Its disappearance was followed by a delay period of 300–1,200 ms at the end of which the subject had to execute a saccade to the memorized location of the briefly flashed target to be rewarded. Finally, the fixation task, shown at the bottom of Figure 1, started with the display of a “fixation” solid white circle at the center of the screen which turned into an “instruction” green solid circle within 200–300 ms. A peripheral target appeared after a variable delay (200–300 ms) and a cursor identical to the one employed in the hand task appeared 300–600 ms later and moved for 500 ms toward the peripheral target. Following the end of the cursor movement and after a variable delay (200–400 ms), the fixation point turned into an open circle similar to the go cue. The subject had to keep its gaze fixed for an additional 500 ms at the end of which it received its reward.

### Data recording

We recorded extracellularly the discharge of single units of the periarcuate cortex of 3 hemispheres contralateral to the moving hand. To this end we used single glass coated tungsten electrodes (AlphaOmega, Nazareth, Israel), of 0.8–1.2 Mohm impedance (measured at 1 kHz frequency), that we slowly lowered through the dura and the cortical gray matter with the help of a hydraulic micromanipulator (Trent-Wells, Coulterville, CA) that was firmly secured on the recording chamber. Electrode signals were amplified (x10,000; Bak Electronics, Inc., Mt. Airy, MD), filtered (band-pass 2 Hz–10 kHz) and digitized with an A/D converter (CED micro1401-3, Cambridge, UK) at a sampling rate of 20 kHz and stored on disk for off-line analysis.

### Analysis

We used the Spike2 version 5 software (CED, Cambridge, UK) to detect and sort spikes. Spikes belonging to different cells that were recorded simultaneously were separated off-line with the help of the clustering and template matching routines of Spike2. Further, we used custom scripts written in the MATLAB (MathWorks, Natick, MA) environment to analyze spike trains and eye/hand trajectories. Movement onset was defined automatically as the moment when velocity exceeded 20 deg/sec for saccades (Moschovakis et al., 1998a), and 1% of maximal velocity for hand movements (Sainburg et al., 1999). Eye velocity (in deg/s) was obtained by numerical differentiation and smoothing of the instantaneous eye position trace. Hand velocity (also in deg/s) was obtained by numerical differentiation and smoothing of the instantaneous joystick controlled position of the cursor on the screen. Spike trains were transformed into a smoothed version of the instantaneous firing rate function, R_n_ = 5/(t_n+3_–t_n−2_). For each spike, occurring at time t_n_, we first obtained the running average of five consecutive inter-spike intervals, (t_n+3_–t_n−2_)/5, two before and three after the spike in question. R_n_ was obtained from the inversion of this running average inter-spike interval. We aligned the instantaneous firing rate functions of single trials on movement onset and evaluated the median value from all the trials to the same target. Then the instantaneous firing rate function was normalized to express it as multiples of standard deviation (SD) away from the mean rate-μ-of background discharge during the reference period (defined as the period ending with the go cue and starting 300 ms before its appearance). Discharge onset for a particular trial was defined as the time when instantaneous firing rate exceeded background discharge by 2 SD. Onsets were visually checked and corrected when necessary. Because for many of the neurons we describe, discharge onset varies with movement direction, the discharge latency we refer to in this study is the one obtained for movements in the neuron's preferred direction.

To be included in this study the onset of a neuron's discharge had to precede the onset of saccades as well as the onset of the hand movements. Furthermore, the intensity of its discharge during the movement epoch (ME) had to exceed significantly (unpaired *t*-test) that of its background discharge during the reference period (300 ms before and until the appearance of the go cue). Since the eyes and the hand did not move simultaneously and to minimize contamination with post-movement activity, we defined the hand related ME as the time interval from 60 ms before until 20 ms after the onset of the hand movement in hand and eye/hand trials. Similarly, we defined the saccade related ME as the interval from 30 ms before until 30 ms after the onset of saccades in eye and eye/hand trials. Moreover, to avoid contamination with delay period activity we also subtracted the mean firing rate during the reference epoch.

Each cell's directional tuning was determined from the goodness of fit of its firing rate to a circular Gaussian (von Mises) distribution. Briefly, we, first, calculated the mean firing rate of all trials during movement of each effector toward each target. The firing rate (R) above baseline activity (mean rate in the period 300 ms before the go cue), was fit with the expression $A\frac{e^{\text{kcos}\left(\text{x}-\text{μ}\right)}}{2\pi I_{o}\left(k\right)}$ where A is a scaling factor, μ the unit's preferred direction, *k* a measure of field width defined as ^2^${/}_{\sqrt{k}}\;$ and *I*_*o*_(*k*) the modified Bessel function of order 0. Unpaired *t*-test between the discharge during the ME (defined in the previous paragraph) and the baseline discharge was used to decide if neuron discharge increased for movements of each effector to each target position. We employed the Watson-Williams test, to evaluate if a cell had a different preferred direction for eye and hand movements and the Rayleigh test to check if the preferred directions were uniformly distributed in a population of neurons. Both tests were part the circular statistics toolbox (Berens, 2009).

To obtain a measure of the duration of neuron discharge, we estimated its half-width defined as the time period between the first rise of instantaneous firing rate above values higher than half way between baseline and peak rate and the first drop below the same value after reaching the peak. The duration of discharge of the neuron for each effector was defined as the mean value of the durations of discharges that significantly exceeded baseline discharge (as indicated with a *t*-test). The peak rate of discharge within this period of time was also measured to analyze, on a trial by trial basis, its relationship with the peak velocity of movements toward the preferred target and, depending on field width, neighboring targets as well. The targets selected were always at the same eccentricity. To assess the temporal relationship between the onset of the discharge and the onset of effector movement we used linear regression and tests of homoscedasticity (Bartlett's and Kepner-Randles tests). The null hypothesis in the Kepner-Randles test is that the data is distributed in a symmetric bivariate manner and the test detects unequal marginal scales. It has the advantage of being non-parametric and does not depend on the value of the mean. A fairly detailed description of the algorithms used in it can be found in Kutz et al. (2003). For *t*-test/ANOVA, Wilcoxon, Bartlett's test, linear regression, and curve fitting we used functions contained in the MATLAB statistical toolbox.

### Track reconstruction and neuron location

We made small injections of Biotinylated Dextran Amine (10 kDa) and placed electrolytic lesions in the right hemisphere of one of the monkeys we studied shortly before its perfusion. The subject was euthanized with an overdose of pentobarbital and perfused with saline followed by a buffered solution of formaldehyde, glutaraldehyde and picric acid. After the end of the perfusion, the brain was photographed *in situ* at a plane parallel to that of the recording chamber (30 deg medial and 13 deg caudal). It was then blocked frontally, and cut frontally in 100 micron sections with a vibratome. Selected sections were processed with DAB (Lanciego et al., 1998). To reconstruct tracks we employed a Zeiss microscope equipped with a drawing tube.

### Decoding

Feed-forward artificial neural networks have been used before to decode motor behavior from the discharge pattern of frontal lobe neurons (Hatsopoulos et al., 2004; Ben Hamed et al., 2007). Here, we built a three layer feedforward artificial neural network, to explore if the discharge pattern of Meq cells could be decoded in a way that would allow specification of movement direction as well as of the effector that would execute it. The activation function of each one of its units ($y_{i}^{\;}$) obeyed the expression $tanh\left(\sum w_{ij}x_{j}^{\;}\right)$ where $x_{j}^{\;}$ are the units driving $y_{i}^{\;}$ and *w*_*ij*_ the connection strengths between units $x_{j}^{\;}$ and $y_{i}^{\;}$. The hidden layer comprised 50 units and the connections between them were initially set randomly to values between −1 and 1. The input layer was made of 55 units each one of which represented the average steady-state intensity of discharge of one of the Meq cells of our sample during the “movement” epoch for an eye movement or a hand movement or a coordinated eye-hand movement. The steady-state intensity in question was selected randomly from all relevant trials for movements of the specific effector in a particular direction in which the cell participated. The output layer was chosen for discrete classification of the effector's identity or the movement's direction. It was made of 8 (when the network decoded movement direction) or 3 (when the network decoded effector identity) units. The target output vector consisted of zeros for the wrong choices and one for the correct choice. To determine if the input vector (i.e., the discharge of 55 Meq units) could specify the movement of the correct effector in the right direction we trained our network using Matlab's scale conjugate gradient backpropagation method. We used 60% of the data to train the networks, 5% for validation and 35% to test the networks' performance. To prevent over-fitting, training stopped as soon as performance in the validation passes started deteriorating. Initial weights were shuffled ten times to ensure that the network would not be trapped in a local minimum. It was deemed to respond correctly when it could deduce effector and movement direction from the discharge intensities that were supplied to its input layer. The importance of the information carried by groups of Meq cells (invariant, non-invariant) was assessed by removing a progressively larger number of its members and evaluating network performance after retraining the network.

## Results

We recorded the discharges of 525 neurons from three hemispheres of two rhesus monkeys; 417 of these cells discharged in relation to some aspect of the tasks we used. One hundred forty eight of these neurons displayed phasic or sustained visual responses but did not discharge phasically before and during movements of the eyes and/or the hand and were excluded from further analysis. We also excluded another 89 neurons whose discharge increased only after the onset of the movement. One hundred and eighty cells discharged phasically before and during movements of the eyes and/or the hand. The present study describes in some detail the phasic, movement related responses of 55 of these cells that discharged before and during coordinated movements of the eyes and the hand as well as movements of one effector not accompanied by movements of the other. Because such cells seem to discharge for the movement executed rather than the effector executing it we refer to them as motor equivalence (Meq) neurons. The same cells also discharged vigorously before memory driven saccades, but not during fixation trials. Thirty-three of them discharged in response to the appearance of a visual stimulus while the remaining 22 did not.

**Figure 2:**
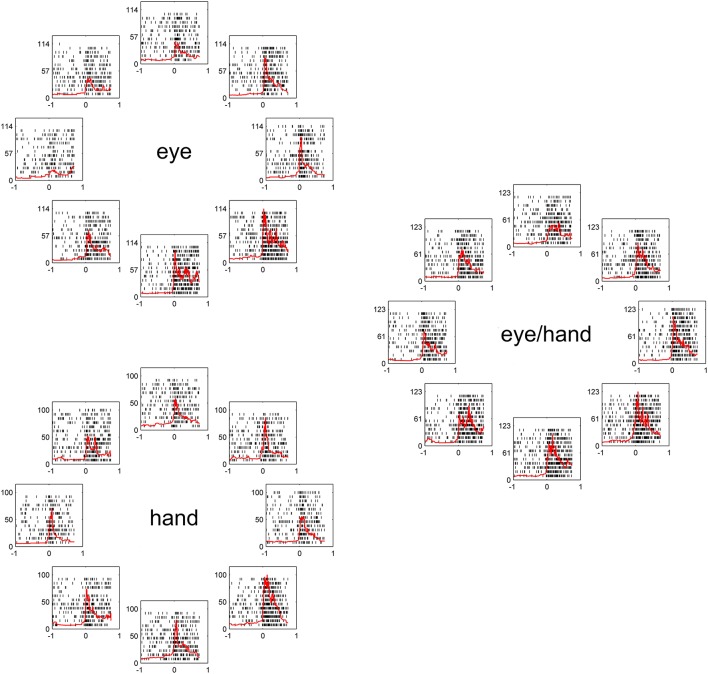
**Examples of the discharge pattern of a Meq cell (L89) for 20° movements to visual targets**. Movements of 8 different directions have been arranged around the periphery of an imaginary circle, separately for eye, hand and eye-hand trials. Red lines indicate instantaneous firing rate and are aligned on movement onset as are the rasters. Abscissa, time (s); Ordinate, instantaneous firing rate (spikes/s).

Figure 2, illustrates a typical example for movements of 20 deg. The movement field of this Meq neuron remained the same irrespective of the effector employed. To examine quantitatively how closely its preferred direction for saccades fits its preferred direction for hand movements, we measured the average firing rate of this neuron for all trials to all targets during the movement epoch. We fit a von Mises distribution to the data for movements in all directions, separately for different amplitudes (10 and 20 deg) and tasks (eye, hand, and eye/hand). The on-direction of the neuron shown in Figure 2, obtained from the von Mises distribution (see Methods), was 246° for saccades, 290° for hand movements and 291° for coordinated movements of the eyes and hand. To determine if the size of the neuron's movement field varied with the effector employed, we compared the values of the parameter ^2^${/}_{\sqrt{k}}\;$ after fitting movement fields with the von Mises distribution as described in the Methods. They did not differ significantly from one task to another measuring 199°, 228°, and 185° for eye, hand and eye/hand tasks, respectively.

**Figure 3:**
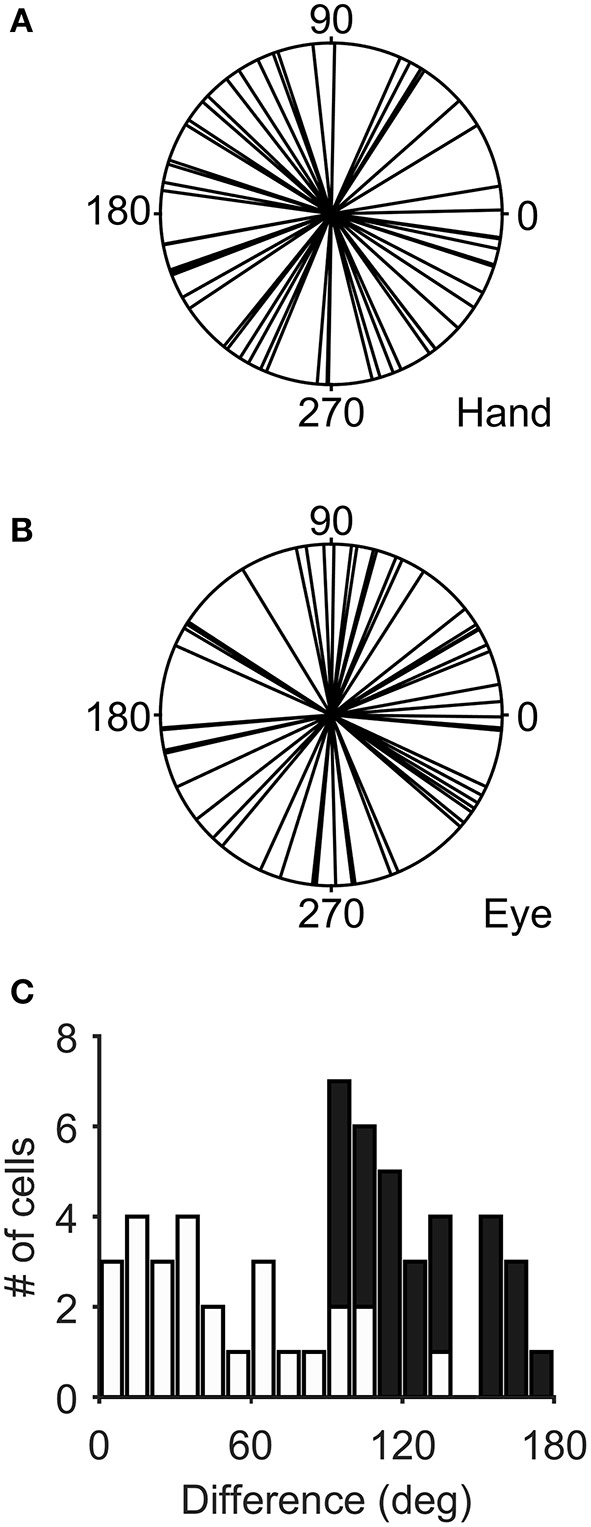
**Distribution of the preferred directions of Meq neurons for hand movements (A)** and saccades **(B)**. The frequency histogram **(C)** illustrates the magnitude of the difference between each neuron's preferred direction for saccades and preferred direction for hand movements. Solid bars indicate statistically significant differences (*p* < 0.05, Watson-Williams).

Figure 3 illustrates the distribution of the preferred directions of the Meq neurons we encountered. They did not display a preference for contraversive or ipsiversive directions for either the eyes or the hand nor did we find evidence of a departure from the uniform distribution for either effector (Raleigh test: *p* = 0.66 for the hand and *p* = 0.3 for the eye). To examine if the on-direction of a neuron for saccades matched its on-direction for hand movements we measured the angular distance of the two. In about half of the neurons (28/55) this did not differ significantly from zero (such as in the case of the neuron illustrated in Figure 2) using the Watson-Williams test. Cells such as these are marked “invariant” in the second column of Table 1, which summarizes the properties of the discharge of the Meq neurons we encountered.

**Table 1:** **Classification of Meq cells according to the effector they prefer (eye, dark; hand, gray)**.

**Cell ID**	**On-direction**	**Intensity**	**Duration**	**Onset**
L36	Invariant	eye	Same	Both
L42A1	Hand	Hand	Same	Both
L42A4	***Mixed***	Hand	Same	Both
L42b	Eye	Same	Same	Both
L45	Hand	Same	Same	Both
L47	Invariant	Same	Same	Both
L52b	Invariant	Same	Eye	Eye
L53	Invariant	Same	Eye	Both
L56	***Mixed***	Same	Same	Eye
L61	***Intermediate***	Same	Same	Eye
L62	***Intermediate***	Same	Same	Both
L63	Hand	Same	Eye	Hand
L76	Hand	Same	***Mixed***	Both
L78	Invariant	Same	Same	Both
L79	Invariant	Eye	Eye	Both
L81	Hand	Same	Same	Eye
L83	Invariant	Same	Eye	Eye
L84	***Intermediate***	Eye	Same	Both
L87	Invariant	Same	Same	Both
L88b	Hand	Same	Hand	Eye
L89	Invariant	Same	Same	Both
L91	Invariant	Same	Eye	Both
L94	***Intermediate***	Hand	Same	Eye
L96	Invariant	Same	***Intermediate***	Both
L97	Eye	Eye	Same	Hand
L98	Invariant	Same	***Mixed***	Both
L100	Invariant	Same	Same	Eye
R2	Invariant	Same	Same	Both
R37	Invariant	Same	Same	Both
R50	Invariant	Same	Same	Both
R62	Eye	Hand	Hand	Both
R202	***Mixed***	Same	Eye	Eye
R204	***Intermediate***	***Intermediate***	Hand	Eye
R211	Eye	Eye	Eye	Both
R216	***Mixed***	Same	***Mixed***	Both
R222	Invariant	Same	Eye	Both
R224	***Intermediate***	Hand	Same	Both
R233	Hand	Same	Same	Hand
R234	Eye	Same	Same	Both
R238	Hand	Same	Same	Eye
R239	Invariant	Same	Same	Eye
R244	Eye	Same	***Intermediate***	Both
R248	Invariant	Hand	Same	Both
R249	Invariant	Hand	Same	Both
R250	Invariant	Same	Eye	Both
R251	Invariant	Hand	Invariant	Both
R333	Eye	Same	***Intermediate***	Both
R337	Invariant	Eye	Eye	Both
R338	Eye	Same	Same	Eye
340	Eye	Same	Eye	Eye
R352	Eye	Same	Same	Both
R353	Invariant	Same	Hand	Both
R354	Invariant	Same	Hand	Eye
R357	Invariant	Same	Same	Both
R358	Invariant	Same	Same	Hand

**Figure 4:**
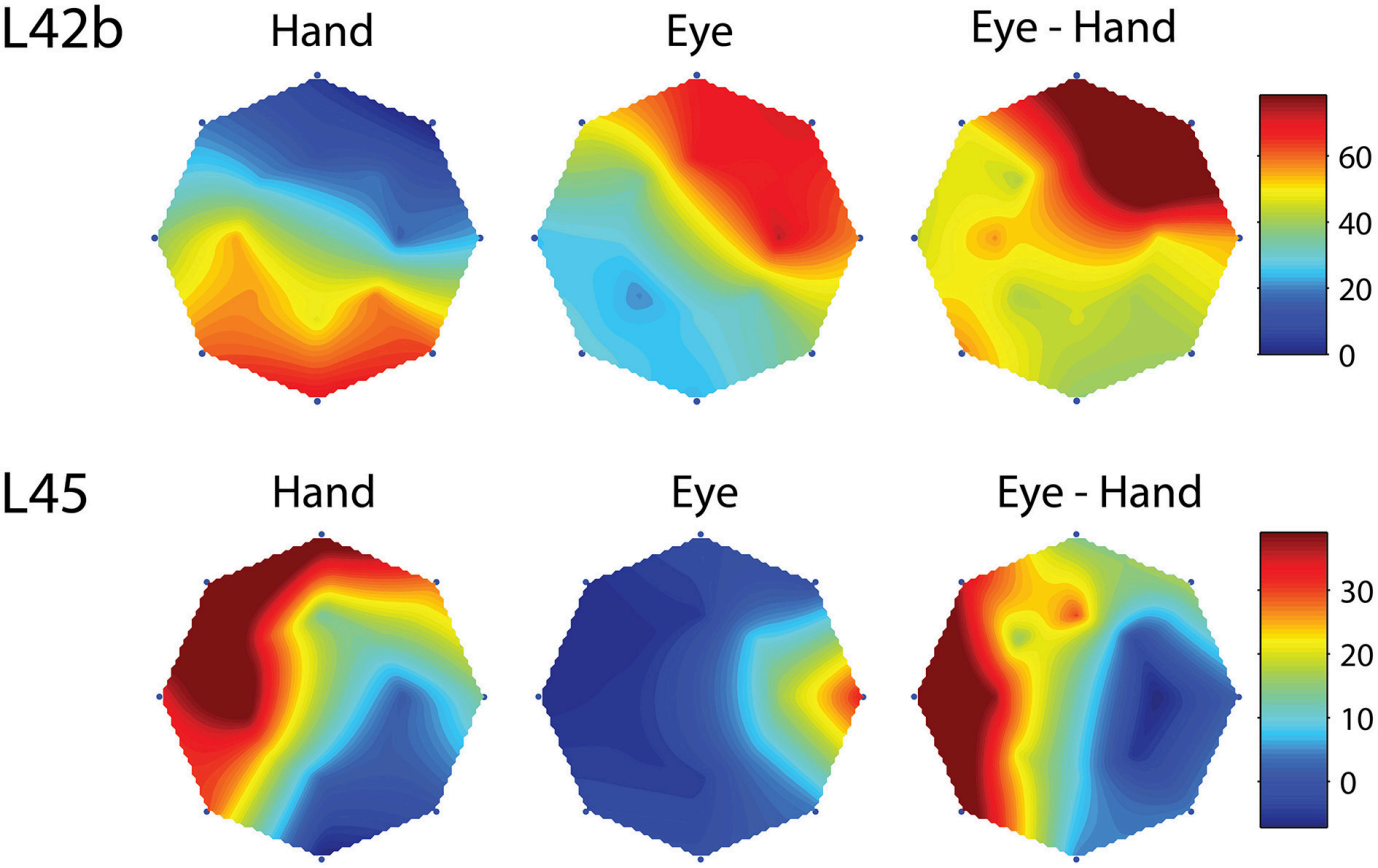
**Examples of movement fields of neurons that prefer one direction for saccades and another for hand movements**. The color scale on the right is proportional to discharge intensity (spikes/s).

**Figure 5:**
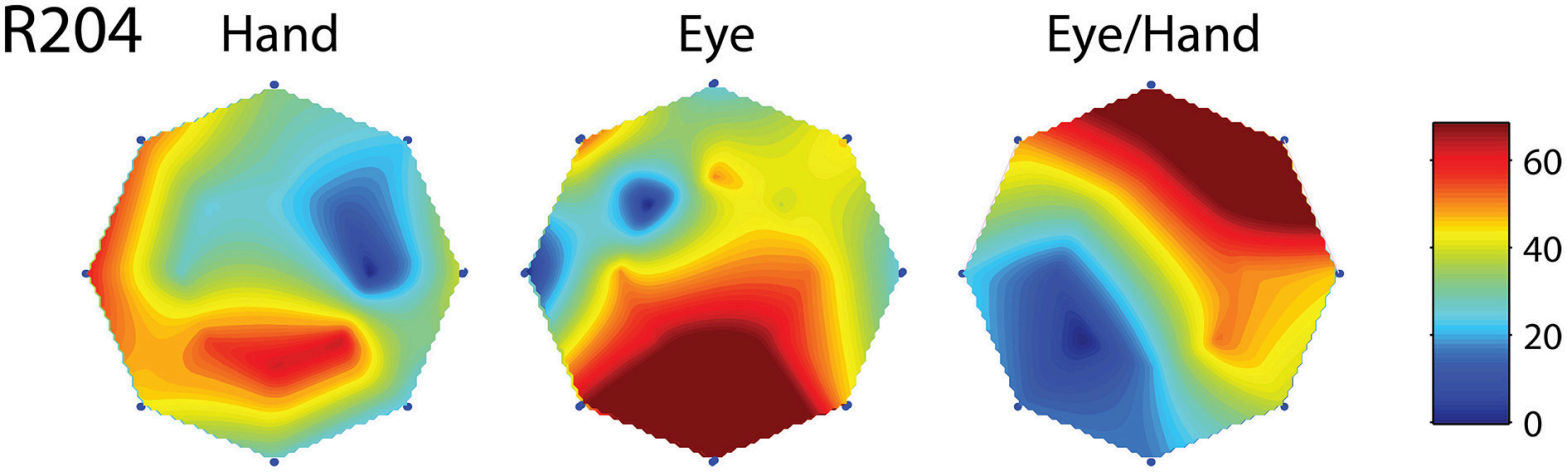
**Movement fields of a neuron (R204) whose on-direction for eye-hand movements did not resemble either its on-direction for hand movements or its on-direction for saccades**. Layout as in Figure 4.

However, this was not the case in the remaining cells (corresponding to the black bars in the frequency histogram of Figure 3C). In these cases, we felt it would be interesting to examine if the on-direction of the neuron for eye/hand movements resembles that for saccades or that for hand movements. In fact, both alternatives were realized as shown in Figure 4. Neuron L42b (Figure 4, top) discharged for downward hand but preferred upward eye movements. It also discharged for upward movements when both the eyes and the hand moved to the same target. Ten of the neurons we encountered displayed a similar preference during coordinated eye/hand movements and are marked “eye” in the second column of Table 1. In contrast cell L45 (Figure 4, bottom) discharged for rightward eye movements but preferred leftward hand movements while it also discharged for leftward coordinated eye-hand movements. A total of 8 neurons discharged in a similar manner in the eye/hand task and are marked “hand” in the “On-direction” column of Table 1. Figure 5 illustrates a neuron whose movement fields for single effector movements did not bear a simple relationship to that constructed from eye/hand movements. It discharged for right-down hand movements and downward eye movements yet it discharged for up-left eye/hand movements. The eye-hand related on-directions of another 5 Meq cells were similar in the sense that they preferred movement directions “intermediate” relative to those for single effector movements and are marked as such in Table 1.

**Figure 6:**
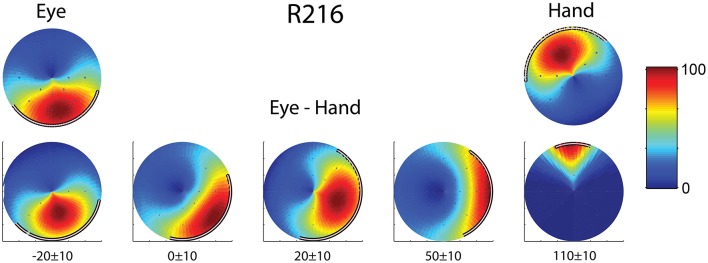
**Example of a neuron (R216) which exhibited dynamic shift of preferred direction during eye/hand movements**. The top row displays the fields for single effector (eyes, left; hand, right) movements. The bottom row shows the gradual transition of the movement field for coordinated eye/hand movements. Values at the bottom indicate the onset and offset of the rasters that provided the data for constructing the field in question. The color scale on the right is proportional to the normalized intensity of discharge.

Finally, we found two neurons whose movement field changed dynamically during coordinated eye/hand trials gradually shifting from one akin to the movement field of saccades earlier in the trial to one resembling the hand movement field later in the same trial. An example is shown in Figure 6 which illustrates the 2D surface fits to the mean rate of discharge of cell R216 during the movement epoch in saccade trials (left) and hand movement trials (right). The surface representing the movement field of the neuron was defined as the product of a Gaussian distribution (for amplitude) and a von Mises distribution (for directions). We employed the least-squares method to find the parameters of the surface that best fit the data. As shown in the top part of Figure 6, the direction of saccades it preferred (downward) clearly differed from that hand movements (upward). The bottom part of Figure 6 illustrates the movement field of this neuron for coordinated eye/hand movements during different time slices of 20 ms duration, starting from 30 ms before saccade onset (leftmost movement field of Figure 6) and ending 120 ms after saccade onset (rightmost movement field). As shown here, the cell starts firing for downward movements (i.e., in the direction preferred by saccades) and ends firing for upward movements (i.e., in the direction preferred by hand movements). Such neurons were marked “mixed” in the “On-direction” column of Table 1.

**Figure 7:**
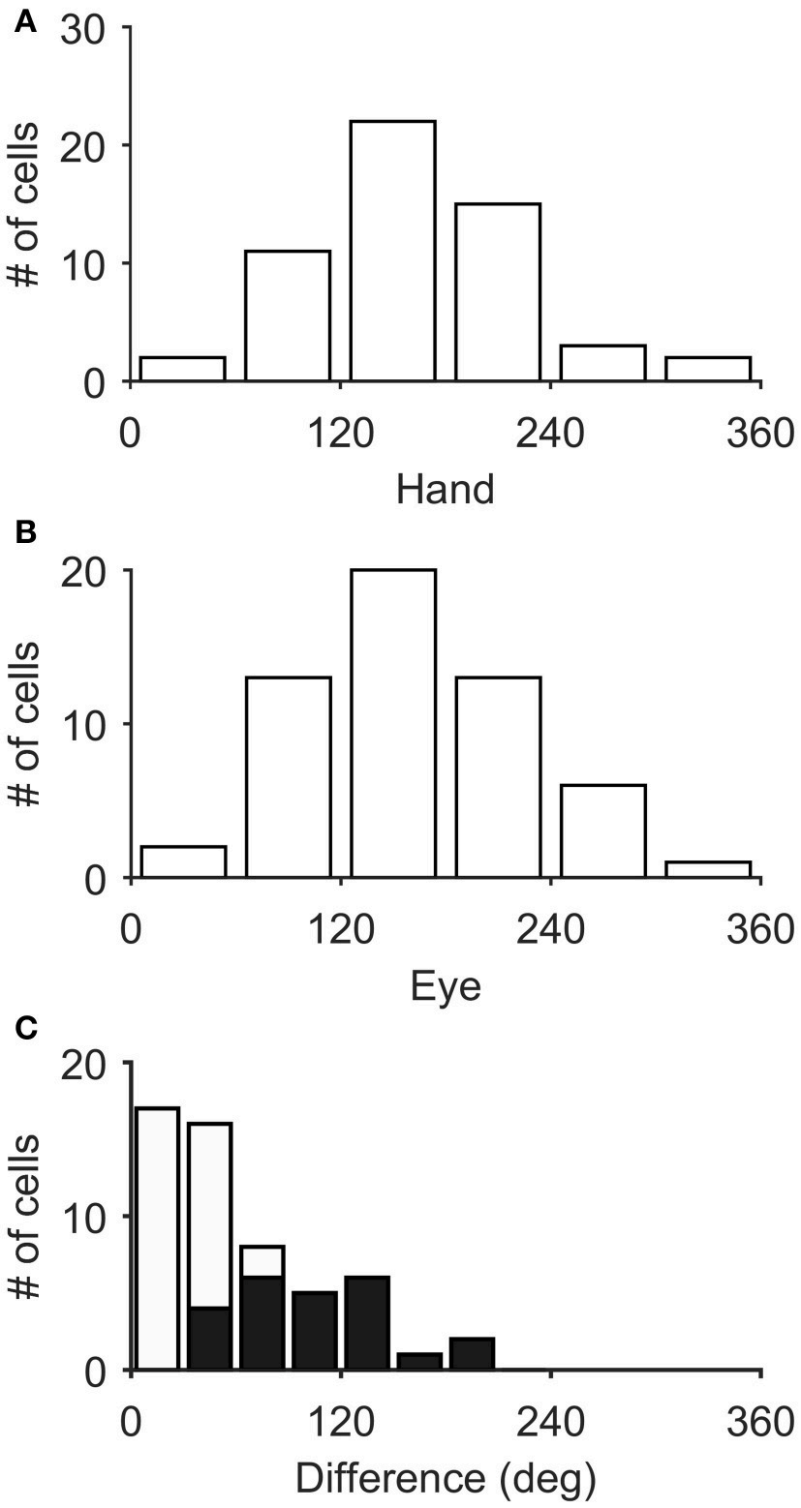
**Distribution of field widths for hand (A)** and eye movements **(B)**. **(C)** Frequency histogram of their differences. Black bars indicate statistically significant values.

The movement fields of Meq neurons were rather wide. They measured 160 ± 62° (mean ± SD) for saccades and 162 ± 65° for hand movements as determined from the 2/$\sqrt{k}$ parameter of the von Mises distribution fitted to them. To obtain an intuitive feeling of their size it is instructive to compare them to the size of the movement field of M1 neurons described by Amirikian and Georgopoulos (2000). The median half-width of 30 M1 cells with symmetric profiles equaled 56° and would obtain a value of 90° for a truly sinusoidal field. Instead the 2/$\sqrt{k}$ parameter of a truly sinusoidal field would equal 117° if one were to fit it with the von Mises distribution. Cells with wide movement fields for saccades did not always display wide movement fields for hand movements and the same was true for cells with narrow movement fields (Figure 7). In fact, the width of the saccade related movement field of 25 Meq neurons was significantly different (Figure 7C, solid) from that of the hand related movement field of the same cells (*p* < 0.05, Bartlett's test).

**Figure 8:**
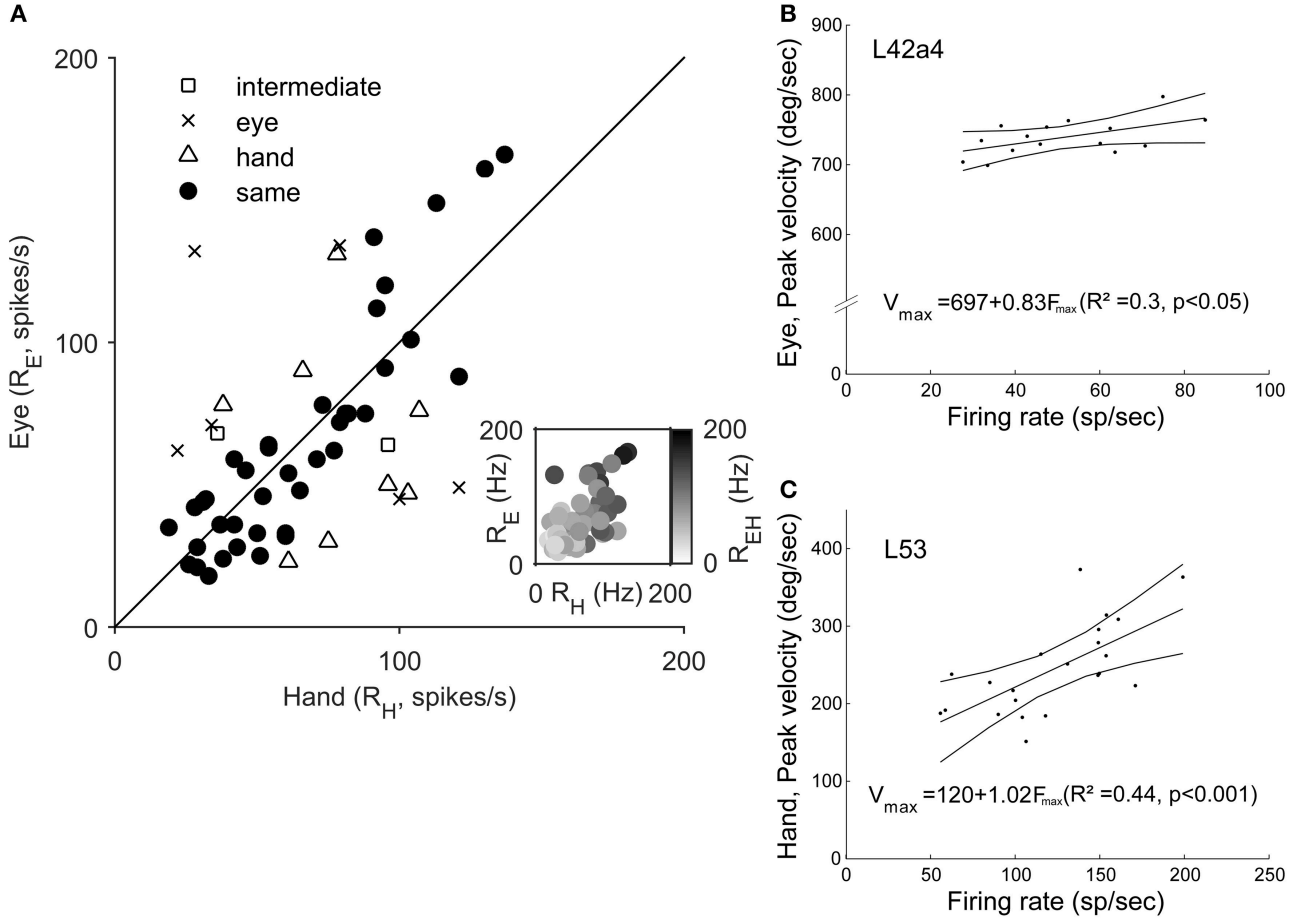
**(A)** Scatter plot of the relationship of Meq neuron rate of discharge for hand movements (R_H_, abscissa) vs. rate of discharge for eye movements (R_E_, ordinate). Each data point is from a different neuron. The straight line is the unity line. Solid circles indicate neurons with similar discharges for eye and hand movements (*p* > 0.05, *t*-test). The inset uses a gray scale to illustrate the relationship between the rate of discharge for coordinated eye-hand movements (EH) and Eye (E) as well as Hand (H) movements. **(B,C)** Examples of the relationship between peak effector velocity (ordinate) and peak discharge rate (abscissa) for two Meq neurons. Lines indicate the linear regression lines (straight), obeying the expressions displayed, and the 95% confidence intervals.

To examine if Meq neurons show a preference for eye or hand movements in terms of firing rate, we measured their mean rate of discharge during the half-width of their phasic discharge, defined as the time period between the first rise of the instantaneous firing rate function to values higher than $\frac{\text{peak rate }-\text{ baseline rate }}{2}$ and the first drop below this value. It could be as low as 18 spikes/s or as high as 166 spikes/s for movements in their preferred direction depending on the neuron and the effector employed. In general, the higher a neuron's rate of discharge for saccades (R_E_) the stronger its rate of discharge for hand movements (R_H_, Figure 8A). The two variables were related through the expression R_E_ = 18 + 0.73R_H_ (*R* = 0.59, *p* < 0.001). The regression of the intensity of discharge for eye-hand movements (R_EH_) onto R_E_ and R_H_ (Figure 8A, inset) was even stronger (R_EH_ = 11.3 + 0.45R_E_ + 0.57R_H_; *R* = 0.81, *p* < 0.001). The eye movement discharges of the majority (39/55) of the Meq cells we studied did not differ significantly from those for hand movements. Such cells lie close to the diagonal and are marked as solid circles in Figure 8A. They are also marked as “same” in the “intensity” column of Table 1. Sixteen neurons displayed significantly stronger discharges (*p* < 0.05, *t*-test) for one of the effectors (half for the eye and the remaining 8 for the hand). In six of them, the discharges during coordinated eye-hand movements were statistically indistinguishable from those during single effector saccades while they were indistinguishable from single effector hand movements in another 8 cells. The intensity of the discharge for eye-hand movements of the remaining 2 neurons (marked intermediate in the “intensity” column of Table 1) did not differ significantly from that during either saccades or hand movements.

The intensity of Meq neuron discharges might be related to a movement related physical variable such as effector velocity. To see if this was the case we plotted peak firing rate during individual trials vs. peak effector velocity for movements of the same amplitude in the preferred direction and the two neighboring ones after subtracting background discharge. Figures 8B,C shows two examples of the good relationship between neuron discharge and movement kinematics. Figure 8B illustrates a neuron (L42a4) whose peak firing rate was significantly related (*p* < 0.05) to peak eye velocity while Figure 8C illustrates a cell (L53) whose peak firing rate was significantly related (*p* < 0.001) to peak hand velocity. Such relationships were rare; peak discharge was significantly correlated to eye velocity in 5 cells, to hand velocity in 7 cells while it was related to both eye and hand velocity in 2 cells.

**Figure 9:**
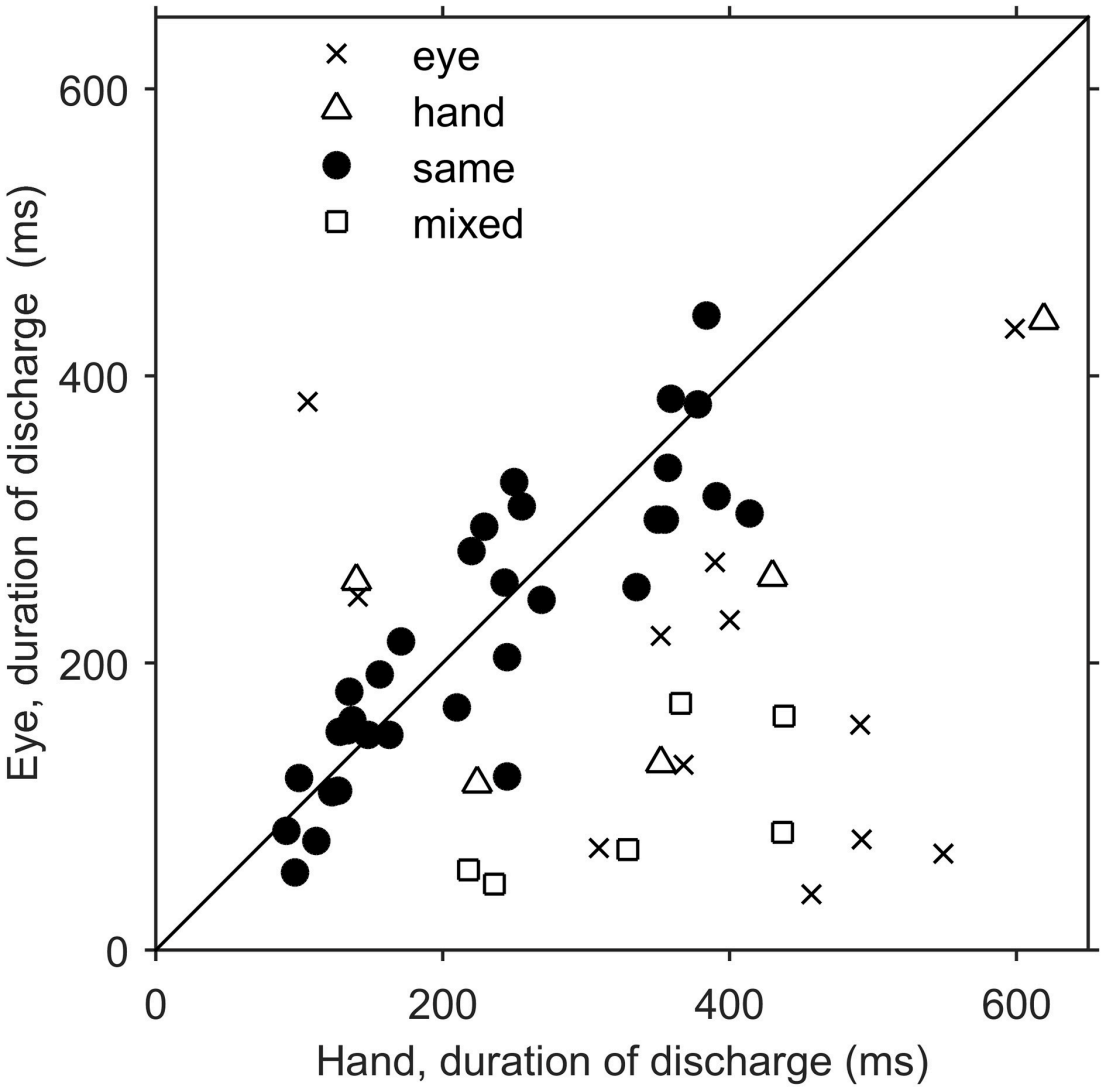
**Scatterplot of the average duration of discharge of Meq neurons in trials when the monkey had to move their hands alone (abscissa) vs. the average duration of discharge of the same neurons in saccade trials (ordinate)**. Each point is from a different neuron. Solid circles indicate neurons with similar durations of discharge for eye and hand movements (*p* > 0.05, *t*-test).

**Figure 10:**
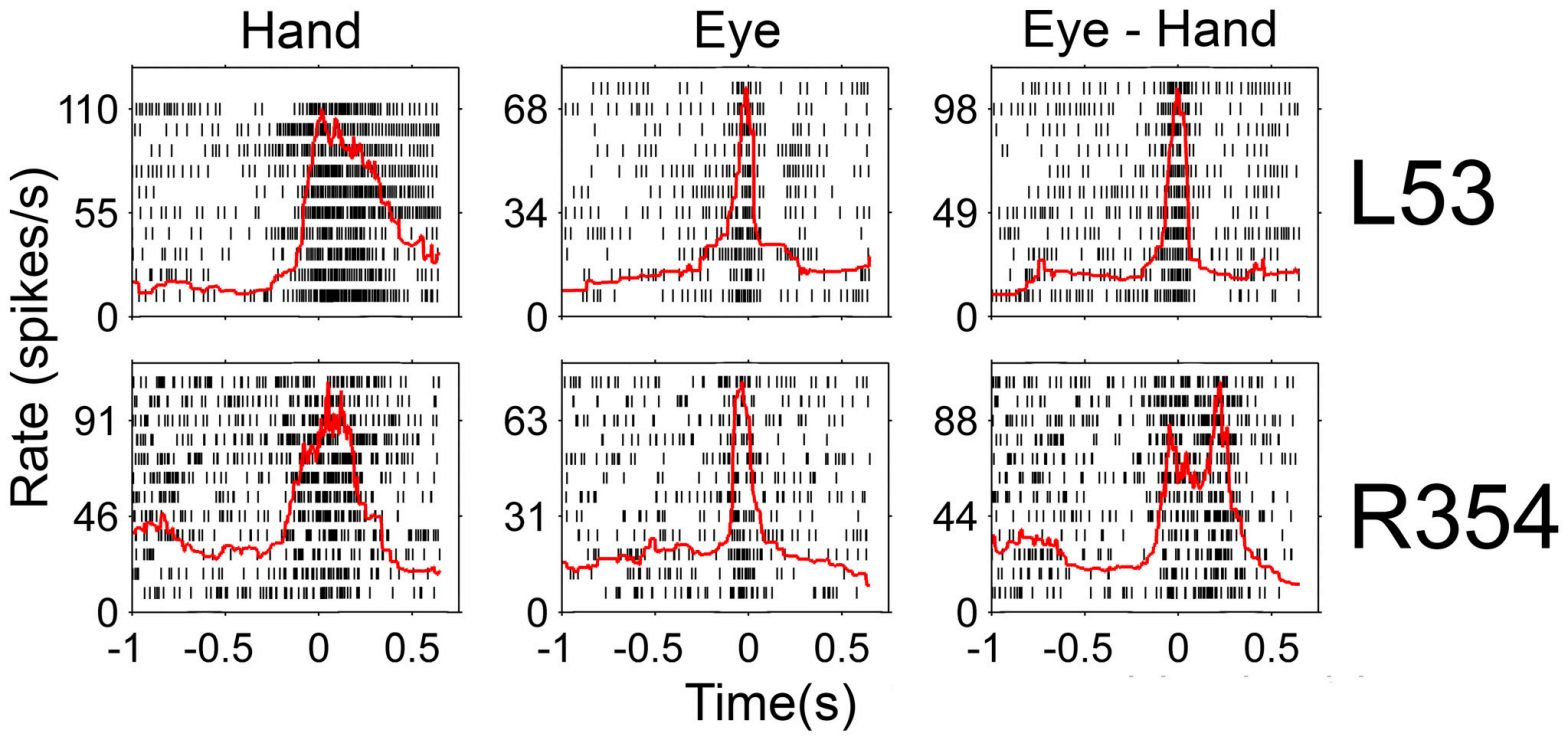
**Examples of neurons whose duration of discharge was not the same for eye and hand movements**. Red lines indicate the instantaneous firing rate and are aligned on movement onset as are the rasters.

Since there was an almost 5-fold difference between the duration of saccades (10°: 45 ms, 20°: 58 ms) and hand movements (10°: 198 ms, 20°: 257 ms) we decided to examine if the duration of the discharge of Meq neurons for saccades differs from that for hand movements. To this end we measured the half-width of the movement related discharge as defined above. Targets eliciting movements not accompanied by statistically significant (unpaired *t*-test) discharges (relative to the background discharge) were discarded as were noisy trials. An indication of discharge duration is obtained from the mean value of the half-widths of all movements to retained targets. As shown in Figure 9, the duration of the discharge of several Meq neurons was equally short or long for hand movements and saccades. These cells (32/55) lie close to the diagonal and are marked as solid circles in Figure 9. They are also marked as “same” in the “duration” column of Table 1. Several other neurons (*N* = 23) did not behave in this manner. With three exceptions (the two x and one open triangle to the left of the diagonal) these exhibited short discharges for saccades and prolonged discharges for hand movements. Given the fact that the difference is worth several hundreds of milliseconds it is meaningful to ask if the duration of the discharge of such a neuron would be short (i.e., saccade like) or long (i.e., hand like) when neuron discharges accompany coordinated movements of the eyes and hand. Figure 10 shows two examples. The top one (cell L53) displayed long discharges for hand movements and short discharges for saccades and also displayed short discharges during coordinated eye/hand movements. Twelve of the Meq cells we encountered discharged in a similar manner and are marked “eye” in the duration column of Table 1. This contrasts the discharge of cell R354 (Figure 10, bottom) which also displayed long discharges for hand movements and short discharges for saccades, but emitted bursts of fairly long duration for coordinated eye/hand movements. Another 5 neurons discharged in a similar manner (marked “hand” in the duration column of Table 1) while the duration of the discharge of 3 cells for eye/hand movements was intermediate relative to that for saccades and hand movements.

**Figure 11:**
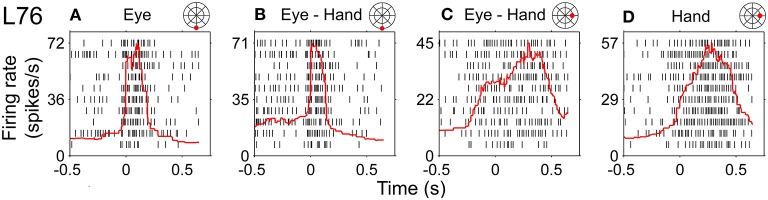
**Example of neuron (L76) whose duration of discharge for coordinated eye-hand movements could be either short (saccade-like) or long (hand-like) depending on the direction of the eye-hand movement. (A,B)** Rasters from downward movements (shown by the red solid circle on the cartoon of directions and amplitudes) aligned onto saccade onset. **(C,D)** Rightward movements aligned onto hand movement onset.

Figure 11 illustrates a somewhat more complex discharge pattern that we observed very infrequently (*N* = 3). As with the neurons described in the previous paragraph, this cell (L76) displayed long discharges for hand movements and short discharges for saccades. It is important to note that the preferred direction of this neuron also differed depending on the effector that was to be moved. It was 265° (i.e., downward) for saccades and 41° (i.e., rightward) for hand movements. In turn, the duration of the discharge of cell L76 for coordinated eye/hand movements depended on the direction of the movement. It was short for downward eye/hand movements (i.e., movements in the direction preferred by the eyes) and long for rightward eye/hand movements (i.e., movements in the direction preferred by the hand). Cells such as this are marked “mixed” in the Duration column of Table 1.

**Figure 12:**
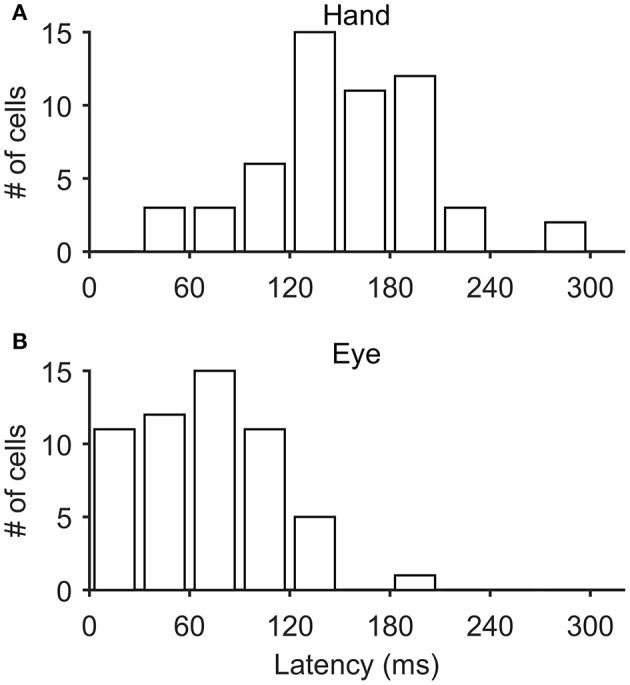
**Frequency histogram of the distribution of latencies of Meq neurons relative to the onset of hand movements (A)** and saccades **(B)**.

In most of the coordinated eye/hand movements we examined, saccade onset preceded the onset of hand movements by 81 ± 33 ms (mean ± SD; range: −41–148 ms). Accordingly, it was not surprising to see that the discharge of Meq cells led the onset of hand movements by 152 ± 51 ms (mean ± SD; range: 40–294 Figure 12A) but led the onset of saccades by only by 70 ± 41 ms (mean ± SD; range: 6–191 Figure 12B). To further assess the temporal relationship between the onset of the discharge of Meq neurons and the onset of effector movement, we measured the latency of the discharge in individual eye/hand trials. If the discharges of Meq neurons determine the onset of movements of the eyes and/or the hand one would expect the earlier or later onset of their discharge to be translated into the earlier or later onset of the movement of the effector they influence. To examine if this is the case for one or both of the effectors, on a trial by trial basis, we aligned all rasters accompanying coordinated eye-hand movements within 45° of the cell's on-direction on the onset of one of the effectors, and examined the correlation between the onset of the discharge and the onset of the movement of the other effector. We defined the onset of the discharge in each trial separately as the point in time when the instantaneous firing rate exceeded the background discharge by 2 SD (see Methods for further details).

**Figure 13:**
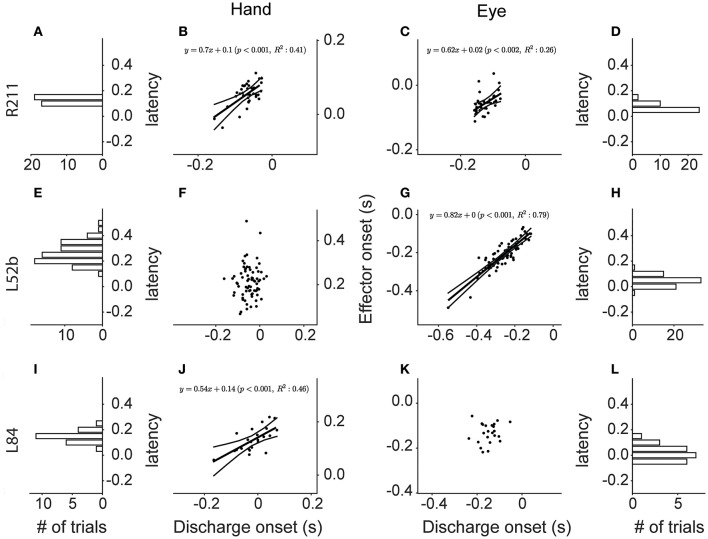
**Scatter plots of the onset of the discharge of Meq neurons (abscissa) vs. the onset of Hand movements (ordinate in B,F,J)** and saccades (ordinate in **C,G,K**) in coordinated eye hand movements. Each point is from a different trial. Lines indicate the linear regression lines (straight), obeying the expression displayed, and the 95% confidence intervals. The frequency histograms show the distribution of the latencies of discharge (behavior onset minus discharge onset, in seconds) relative to the onset of the hand movement **(A,E,I)** or the saccade **(D,H,L)**.

The top row of Figure 13 shows a neuron (R211) whose discharge onset is significantly correlated with the onset of both saccades and hand movements (*p* < 0.002 & *p* < 0.001 respectively). Figure 13B is a scatter plot of the onset of hand movements vs. discharge onset for trials aligned on the onset of saccades. The linear model accounted for a sizable proportion of the variance (41%) of the dependent variable. A statistically significant relationship (*p* < 0.002), albeit weaker (*R*^2^ = 0.26), was also found between the onset of the discharge and the onset of the saccades when the trials were instead aligned on the onset of the hand movement (Figure 13C). The onset of the discharge of another 9 Meq neurons displayed similarly good correlations with the onset of the movement of both effectors (*R*^2^ = 7–41% for hand movements and 8–53% for saccades). Cells such as these are marked “both” in the “Onset” column of Table 1.

Figure 13G is a scatter plot of the onset of saccades vs. discharge onset for trials aligned on the onset of hand movements within 45° of the on direction of cell L52b. The correlation coefficient (0.89) indicates that most of the variance of the dependent variable is accounted for (*p* < 0.001). In this case, aligning the eye-hand trials on saccade onset did not result in a linear relationship between the onset of the hand movement and the onset of the discharge of this cell (Figure 13F). Surprisingly, the majority of Meq neurons in our sample (*N* = 36) displayed such good correlations with the onset of saccades (*R*^2^ = 12–88%) and not the onset of hand movements. One might argue that alignment on the onset of the movement of an effector that is almost perfectly correlated with discharge onset (as is the case in Figure 13G for saccades) would result in the almost perfect alignment of the independent variable as well (in this case the onset of the discharge) and thus its correlation with the onset the movement of the other effector might be poor because the range of values of the independent variable has been restricted excessively. To address this issue, we examined the relationship between discharge onset and movement onset in single effector trials in which the subject was rewarded for moving the hand and not the eye after aligning the trials on the GO signal. Twenty one of the 36 seemingly eye related Meq cells in our sample displayed a statistically significant correlations between discharge onset and hand movement onset (*R*^2^ = 9–55%) in such single effector trials and are also marked “both” in the “Onset” column of Table 1. The onset of the discharge of the remaining 15 Meq cells remained insensitive to the onset of the hand movement even in single effector hand trials; such cells are marked “eye” in the “Onset” column of Table 1.

Finally, the onset of the discharge of some Meq neurons was correlated with the onset of hand movements rather than the onset of saccades. The bottom row of Figure 13 provides an example. Figure 13J is a scatter plot of the onset of hand movements within 45° of the on-direction of cell L84 vs. the onset of its discharges for trials aligned on the onset of saccades. Much of the variance of hand movement onset (*R*^2^ = 46%) is accounted for by the jitter of discharge onset (*p* < 0.001). In the case of neuron L84, aligning the eye-hand trials on the onset of hand movements did not result in a linear relationship between saccade onset and discharge onset (Figure 13K). The discharge of another 8 Meq neurons displayed similar relationships to the onset of effector movement. For reasons outlined in the previous paragraph, in these cases we also examined the relationship between discharge onset and saccade onset in single effector saccade trials after aligning the trials on the GO signal. Five of the 9 seemingly hand related Meq cells displayed statistically significant correlations between the onset of the discharge and the onset of saccades (*R*^2^ = 11–36%) in such single effector trials and are also marked “both” in the “Onset” column of Table 1. The onset of the discharge of the remaining 4 cells remained insensitive to the onset of the saccades in single effector trials and are marked “hand” in the “Onset” column of Table 1. To summarize, the onset of the discharge of the majority of Meq neurons seems better correlated to the onset of saccades than the onset of hand movements.

To assure ourselves that this is the case, we aligned all trials within 45° of a Meq cell's on-direction on the onset of its discharge and constructed the frequency histograms of the latencies of the effector movements they accompany. If the onset of the discharge is better related with the onset of saccades one would expect the frequency histogram of the latency of hand movements to be wider than that of saccades. The opposite would be true if the onset of the discharge was better related to the onset of hand movements. Figures 13I,L are the frequency histograms of the latency of the onset of relevant hand movements and saccades, respectively, which accompany the discharge of neuron L84. The SD is 41 ms for saccade latencies (SD_E_) and 56 ms for hand latencies (SD_H_) again demonstrating that this neuron's discharge is better aligned to the onset of the eye rather than that of the hand movement. For each of the Meq cells we calculated the ratio of SD_E_/SD_H_. It was smaller than one (range: 0.31–0.97) in the 36 Meq cells better related to saccade onset as determined from the jitter analysis described above. For example, the spread of saccade latencies was 3.2 times smaller than that of hand latencies, a highly significant difference (*p* < 0.0002; Bartlett's test), in neuron R238 which occupies one end of this range. The smaller spread of saccade latencies proved statistically significant in 28 of these 36 Meq cells using either Bartlett's (*p* < 0.05–10^−9^) and/or the Kepner-Randles test (*p* < 0.05; see Methods for a short description of this statistic). Conversely, it exceeded one by only 9–34% in the 9 Meq cells better related to hand onset, as determined from the jitter analysis, but these differences did not prove statistically significant. This analysis reinforces our conclusion that the onset of the discharge of Meq cells is better correlated to the onset of saccades than the onset of hand movements.

**Figure 14:**
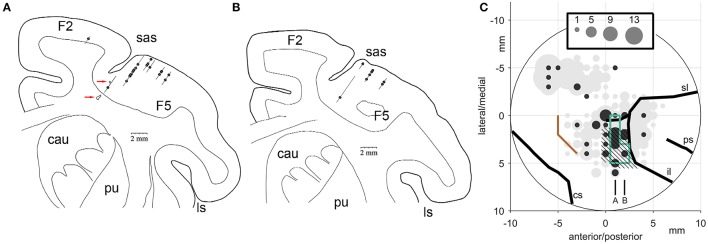
**(A,B)** Frontal sections through the premotor cortex of subject R. Their relative location is indicated in C. **(C)** Location of Meq cells found in the primate periarcuate cortex arranged on a grid centered on the middle of our recording chamber. The area of the discs is proportional to the number of Meq neurons (black discs) or the total number of neurons (gray discs) encountered within 0.5 mm of their center. Data are from 3 hemispheres aligned relative to the smooth pursuit area (enclosed by green lines), area F5 (hatched) and the border between M1 and the premotor cortex (orange). Red arrows point to electrolytic lesions placed in subject R just prior to perfusion to anchor the relative location of electrode tracks. Abbreviations: cs, central sulcus; il, inferior limb of the arcuate sulcus; ps, principal sulcus; sl, superior limb of the arcuate sulcus.

Figure 14 illustrates the number of the Meq neurons we found in different parts of the periarcuate cortex, shown as solid circles the diameter of which is proportional to the number of the Meq cells encountered within 1 mm of its center, juxtaposed to the total number of neurons encountered in the same region (gray circles). Figure 14A illustrates portions of tracks found within 500 microns of the illustrated frontal section which passed 22 mm in front of the interaural line of subject R. A second section one mm rostral the first is also illustrated (Figure 14B). Besides placing small electrolytic lesions (Figure 14A, red arrows) we anchored our observations on the medial and caudal borders of area F5 (Figure 14C, hatched) which was mapped with the help of single cell recording for the needs of an experiment focusing on mirror neurons and in which our subjects also participated. They were also anchored on the borders of the smooth pursuit area (Figure 14C, green lines) that were mapped in this as well as subject L with the help of electrical microstimulation. As shown in Figure 14C, the majority of the Meq neurons in our sample were found in or near the mirror neuron and the smooth pursuit region of the caudal bank of the AS (*N* = 41). We also wished to examine if there are Meq cells outside this relatively restricted area of the AS. We were able to find them more caudally in the premotor cortex, 8 dorsal and another 3 ventral to the spur, while 2 were found intermingled with FEF cells in front of the AS.

## Discussion

The present report describes the response properties of cells located in the premotor cortex and discharging phasically for saccades, hand movements and coordinated movements of the eyes and hand. The relatively high rate of their discharges started before the onset of movement of both effectors and could therefore drive either. To refer to these cells we use Lashley's (1930) term “motor equivalence,” to emphasize the notion that they could encode an abstract, effector invariant form of the vector of desired effector displacement. In contrast to Lashley who used the concept “equivalence of motor responses” as an argument against what he called “the doctrine of the specialization of nervous elements,” we are of the opinion that even the execution of equivalent motor acts can rely on specialized neural substrates.

### Comparison to previous studies

This is not the first report to describe cells of the premotor cortex whose phasic discharges accompany movements of the eyes as well as movements of the hand. Mann et al. (1988) found such cells in the SEF and used the term “motor equivalent” to refer to their responses. Fujii et al. (2002) also found them in the SEF, as well as in the pre-SMA and the SMA and provided illustrations of typical examples in Figure 4C (a pre-SMA neuron) and Figure 5C (SEF neuron). Although, not as often, they were encountered in the dorsal premotor cortex (PMd) as well (Fujii et al., 2000). Nor are effector independent discharges of premotor cortex neurons limited to eye-hand coordination; the PMd contains cells discharging for movement direction whether the effector is the ipsilateral or the contralateral arm (Cisek et al., 2003) while the PMv contains cells discharging for grasps whether executed by the ipsilateral or the contralateral hand (Rizzolatti et al., 1988).

The responses of Meq neurons are not lateralized in the sense that their preferred directions were as likely to be ipsiversive as contraversive. Since these cells discharge phasically for both saccades and hand movements it is reasonable to ask if their movement field for saccades resembles their hand movement related one. This question was asked before regarding cells of area PEc which are tuned to particular directions of reaching and saccades (Battaglia-Mayer et al., 2001). These authors determined the preferred directions of parietal cells during several different epochs and coined the term *global tuning field* (*GTF*) to indicate that they all cluster within a limited sector of space, the GTF (Battaglia-Mayer et al., 2006). As with our Meq neurons, the GTF of PEc cells are isotropically distributed in space. A small number of Meq like cells may have been found in the parietal reach region (PRR). Of 206 PRR cells studied, 29% (*N* = 59) had saccade related activity but it seldom preceded saccades (14/206 = 7%). The preferred directions of reach related discharges were aligned to those of saccade related ones (Snyder, 2000).

The on-direction of the hand related discharges of about half of the Meq cells did not differ significantly from that for saccades. The same is true of the bimanual cells of the PMd; the preferred direction of about half (16/29 = 55%) of them changed little when the task was performed with the other hand (Cisek et al., 2003). It is also consistent with the observations of Pesaran et al. (2010) who studied the delay period activity of PMd neurons. The rather small (19 deg) mean difference they found between on-directions in trials involving saccades and trials involving hand movements led them to conclude that their preferred directions tend to align. However, only half of the Meq neurons we encountered do this. The on-direction of the hand related discharge of the remaining Meq neurons differed considerably from that for saccades. Such cells may in fact have been seen before as well. For example, Figure 3A of Pesaran et al. (2010) illustrates a neuron whose phasic discharges accompanied up-left reaches and down-right saccades. Also, Figure 2 of Battaglia-Mayer et al. (2006) illustrates a cell whose movement epoch discharges increase for left-up hand movements and down-right saccades while cells of the premotor cortex are known to lack preferred direction constancy across target distances and speeds (Churchland et al., 2006). Finally, in agreement with previous observations, the on-direction of a small number of Meq cells was found to vary with time. Neurons of the premotor cortex have been shown to display a certain preferred direction during the instruction or reaction epoch and a considerably different one during the movement epoch (Suminski et al., 2015). We demonstrate that such shifts of preferred direction can happen within the movement epoch of Meq neurons; their preferred direction can face one way early in the movement and in a different one late in the movement. Such shifts of the perimovement discharges have been shown for some M1 neurons (Churchland and Shenoy, 2007). In their Figure 12 these authors illustrated a neuron whose preferred direction was almost directly rightward just before movement onset (Figure 12A), it turned almost directly leftward 100 ms later (Figure 12B), and became rightward again when another 100 ms had elapsed (Figure 12C).

The duration and the intensity of the burst of Meq neurons for one of the effectors did not always match that for the other. Concordance or dissonance was not a trait of distinct subpopulations of Meq neurons; cells that were effector invariant in some properties (e.g., intensity) might discriminate between effectors when another parameter (e.g., duration) was considered. In fact, the single most important conclusion to be drawn from Table 1 is that no Meq cell showed a preference for one of the effectors (eye or hand) across the board of the variables studied. As expected of cells that could influence the time course of movements, the maximal firing rate of Meq neurons could be significantly correlated to peak velocity, albeit for a rather small number of cells; that of the eyes in 5 cells, of the hand in 7 cells and of both eye and hand in 2 cells. More generally, since Meq cells burst for both saccades and hand movements one might expect the intensity (R_EH_) of the burst of spikes for coordinated eye/hand movements to equal the sum of the intensities of their bursts for single effector movements (R_E_ for saccades and R_H_ for hand movements). This was indeed the case for some of the cells in our sample but again their number was small (8/55). Instead, their bursts were about 50% weaker than expected and followed a trend summarized in the expression R_EH_ = 11.3 + 0.57R_H_ + 0.45R_E_ (*r* = 0.81, *p* < 0.001). Hagan et al. (2012) posed a similar question regarding the delay period discharges of LIP neurons, namely whether the activity of individual neurons increased or decreased when a reach accompanied a saccade. To address this issue they studied 55 LIP neurons displaying spatially selective responses in rhesus macaques performing memory-guided saccade or saccade-reach tasks. As with our Meq neurons, their data indicate that the higher a cell fired for saccades the higher it fired for coordinated eye-hand movements as well (Figure 3E of Hagan et al., 2012). These authors also found that nearly half of their cells [26/55 (47%)] showed a significant difference between the two tasks, 12 of the 26 (46%) emitting higher discharges for saccades unaccompanied by reaches while the remaining (54%) emitted higher discharges for coordinated eye-hand movements. In contrast, only 12 Meq cells showed a statistically significant difference in activity between the eye and eye-hand tasks; 9 of them increased and 3 decreased their discharge for coordinated eye-hand movements. When the eye-hand related discharge of our Meq cells is compared to their hand related one, again the higher a cell fires for hand movements the higher it fires for coordinated eye-hand movements (*R*^2^ = 0.53, *p* < 5 × 10^−10^). Again, only 13 Meq cells showed a statistically significant difference in activity between the hand and eye-hand tasks; 10 cells fired more and 3 less for coordinated eye-hand movements. The excess discharges shown by the 10 Meq cells for coordinated eye hand movements are reminiscent of the findings of Jouffrais and Boussaoud (1999) who demonstrated that the intensity of discharge of PMd cells increases, albeit for long periods of time, when a saccade precedes the hand movement to the target but not when it does not.

The duration of the movement related burst of the majority (31/55 = 56%) of the Meq neurons we encountered did not depend on the effector executing the movement; cells that discharged for a short (long) period of time for saccades also discharged for a short (long) period of time for hand movements. With three exceptions, the bursts of the remaining Meq cells were brief when they accompanied saccades and prolonged when they accompanied hand movements. This is consistent with the duration of the hand (long) and saccade (short) related bursts of the superior colliculus neurons illustrated by Werner et al. (1997a) and of the PMd neurons illustrated by Fujii et al. (2000). The duration of bursts for coordinated eye hand movements was more difficult to predict. Since saccades precede hand movements by 81 ms on the average, one would expect their eye-hand related bursts to last for at least as long as the time distance between eye and hand movement onsets plus the duration of their hand movement related discharges. In fact, it was usually shorter than that of bursts accompanying isolated hand movements and in some cases mixed, in the sense that the cells emitted short bursts for eye/hand movements in some directions and long bursts for eye/hand movements in other directions.

Meq-like cells that burst for both eye and hand movements need not be confined to the premotor or parietal cortex. Previous studies have shown that the visual, saccadic and/or preparatory activity of more than half of the FEF neurons can be modulated by hand position, whether the hand is visible or invisible (Thura et al., 2011). Moreover, the FEF did not show any significant effector preference when fMRI was used to examine if cortical areas would be preferentially activated for saccades or reaches (Levy et al., 2007) while extracellular records obtained from primate FEF cells showed at most a modest preference for saccades in a study focusing on tonic delay period discharges (Lawrence and Snyder, 2006). However, to our knowledge there are no descriptions of saccade related FEF neurons discharging phasically for movements of the hand. In our exploration of the FEF region, we encountered only 2 Meq neurons. Their discharge was not remarkable when compared to that of Meq cells of the premotor cortex. They preferred rightward movements of the hand but leftward or downward movements of the eye. The onset of their discharge preceded saccades by about 85 ms and hand movements by about 130 ms and was well correlated to saccade onset in both cells and to the onset of hand movements in one of them. Actually, it is a subcortical region, the superior colliculus (SC) that contains cells with clear Meq-like responses. Werner (1993) and Werner et al. (1997b) Asked monkeys to look at visual targets and after a period of time that could last for hundreds of milliseconds, reach for them. They found 31 cells (about 10% of the total encountered) that resemble our Meq cells, many of them inside the SC and the remaining in the underlying reticular formation. Burst duration depended on the effector executing the movement, and could be short (eye movement) or long (hand movement). As with our Meq neurons, a large percentage (24/31) of the SC cells responded to visual stimulation as well. The anatomical connections between area 6 and the SC (Leichnetz, 1981; Fries, 1984, 1985) could account for the presence of Meq cells in both areas.

### Does a single command drive the eyes and the hand?

When looking at an object and manipulating it by hand, saccades and hand movements must be coordinated in space and time. Early studies raised the possibility that the two effectors are controlled by a common command (summarized in Kattoulas et al., 2008). During coordinated movements of the eyes and the hand to the same target, our monkeys first executed a saccade and then a hand movement within a time interval of about 80 ms. Somewhat longer intervals have been seen in monkeys executing coordinated saccades and reaches from a central fixation point to a peripheral target in an overlap paradigm (150 ms; Rogal et al., 1985) or to an odd-colored target among distractors (235 ms in one monkey and 139 ms in another; Song and McPeek, 2009). The extremely short standard deviations these authors reported (1.1 and 1.2, respectively) are indicative of the stereotyped coupling of the two effectors. Intervals roughly equal to 100 ms have been found in human subjects (Herman et al., 1981; Lünenburger et al., 2000; Boucher et al., 2007). An interval that remained constant at 84 ms for a variety of tasks (step, gap, memory, scanning, and antisaccade) was reported by Sailer et al. (2000). Interestingly, Fisk and Goodale (1985) took advantage of the fact that contraversive arm movements start some 40–50 ms after ipsiversive ones and that movements of the right arm precede those of the left to examine how well the eyes are yoked to the hand. If well yoked then rightward saccades accompanying right-handed ipsiversive reaches should be initiated earlier than right-going saccades accompanying left-handed reaches to the same target. This was indeed the case, the difference being nearly 50 ms (*t* = 2.5, *p* < 0.05), even though in both cases the eyes were moving in the same direction (Fisk and Goodale, 1985). The notion that movements of the eyes and hand are coupled (at least temporally) is supported by one of the symptoms of the parietal syndrome, directional hypokinesia, which leads to an increase of the reaction time of both hand movements (Faugier-Grimaud et al., 1985; Mattingley et al., 1994) and saccades (Girotti et al., 1983) to contralateral targets. The same is true of the monkey where LIP lesions delay the onset of both the saccadic and the reaching components of coordinated eye-hand movements (Yttri et al., 2013).

A similar conclusion can be reached in studies of the correlation between the reaction times of the eyes and the hand. These have been variously reported as very low or insignificant (Mather and Fisk, 1985; Bekkering et al., 1994), poor (*r* < 0.4; Biguer et al., 1982; Frens and Erkelens, 1991), middling (≈0.5; Prablanc et al., 1979a; Gielen et al., 1984; Fischer and Rogal, 1986; Sailer et al., 2000; Suzuki et al., 2008) or high (>0.6; Herman et al., 1981; Fischer and Rogal, 1986; Frens and Erkelens, 1991; Suzuki et al., 2008; Yttri et al., 2013). Before we examine possible causes of this impressive variety, it is instructive to note that even low correlation coefficients can be highly significant. For example, Biguer et al. (1982) measured the latency of eye, head and hand movements toward targets. Although, correlation coefficients were below 0.5 they were significant at the 0.001 level.

The correlation coefficients that have been reported seem to be sensitive to subject idiosyncrasies and task design. For example, in a single study they were found to range between 0.16 and 0.62 (*p* < 0.01) depending on the subject and the test (Boucher et al., 2007). Also, low (or absent) correlation on a trial by trial basis could be due to the severe restriction of the range of the independent variable (saccade latency) rather than independent commands delivered to the eyes and hand. This could account for the drop in correlation coefficients between saccade latencies and reach latencies from 0.93 (overlap task) to 0.5 (gap task) along with the range of saccade latencies, from 180 to 430 ms in the overlap task to between 100 and 170 ms in the gap task (Fischer and Rogal, 1986). The same is true of the insignificant correlation found in the study of Bekkering et al. (1994) which emphasized short hand reaction times, atypically shorter than those of saccades. A considerable reduction of the latency of reaching movements together with that of saccades has been shown in gap trials for human subjects (Lünenburger et al., 2000; Sailer et al., 2000; Gribble et al., 2002) and monkeys (Rogal et al., 1985). Along with these, the relatively high correlations (0.41–0.88) reported by Gribble et al. (2002) for overlap trials dropped to 0.22–0.68 in gap trials (*p* < 0.01 in all cases). In turn, correlations increase in tasks that prolong reaction times such as those directed to auditory (Mather and Fisk, 1985) or somatosensory (Neggers and Bekkering, 1999) stimuli or employing memory and antisaccade/antireach tasks (Sailer et al., 2000).

In the present study the correlation coefficient of the relationship between the reaction time of saccades and hand movements on a trial by trial basis was 0.42 in 3,540 individual trials of one subject and 0.49 in 4,329 trials of the other (the probability that this might be due to chance was very close to zero in both monkeys). These values agree well with those from two previous reports for the monkey (0.35–0.45; Yttri et al., 2014; 0.3–0.5; Battaglia-Mayer et al., 2013). Moreover, our study demonstrates that the onset of Meq neuron bursts is tightly coupled to movement onset and could thus be causally relevant for movement execution. Assuming that two causal chains start from the same central process (e.g., Meq cells) and lead to eye movements on the one hand and hand movements on the other, moderate correlations between the onset of the central process and the onset of the movement of each separate effector (in the range of 0.7) would result in correlations between effector onsets that do not exceed 0.5 (≈0.7^2^).

Common representation of desired eye and hand movements is also suggested by the spatial coupling of movements of the two effectors. Both are affected in similar ways by cerebellar ataxia (Engel et al., 2002), visual illusions (Soechting et al., 2001), stimulus modality (Mather and Fisk, 1985), distractors (Sailer et al., 2002a), saccadic adaptation (Bekkering et al., 1995), and manipulation of the flow of information through the saccadic system (Nemire and Bridgeman, 1987). For example, when subjects were asked to look and point with their unseen hand to the same target while the eyes and hand started from different positions such that the eye had to travel a longer distance, hand amplitudes were found to increase with saccadic amplitudes (van Donkelaar, 1997). Also, the size of saccades to a target depends not only on the distance between target and fixation point but also on the presence of a nearby distractor. In cases such as this, saccades often land in between target and distractor. This spatial averaging, “global effect” (Findlay, 1982; Kapoula, 1985) has been shown to simultaneously affect both saccades and hand movements. Using a distractor less eccentric than the target, leads to smaller movements of both the eyes and the hand. Conversely, using a distractor more eccentric than the target, leads to bigger movements of both the eyes and the hand (Sailer et al., 2002a,b). Common spatial representation of desired eye and hand movements is also suggested by the fact that the responses of the eyes to double-step targets resemble those of the hand (Gielen et al., 1984). Nor were the eyes and hand ever seen to move in opposite directions (at least initially) when the two targets are presented in opposite sides of the fixation point. Further, adaptation of the saccadic system transfers to the hand moving system (Bekkering et al., 1995). These authors asked subjects to look at and point (with the help of a hand held device) to a target appearing on a cathode ray tube and which jumped 2 deg back toward the fixation point near the onset of the saccade. Initially the subjects overshot the target and needed to execute a second, corrective saccade, to acquire it. After a few trials the saccade was adapted and its smaller size drove the eyes directly to the final position of the target. The hand movement was also adapted showing similarly shortened amplitudes. Finally, spatial coupling of saccade and reaching movements is suggested by a case of “magnetic misreaching” displayed by a patient who suffered from bilateral parietal lobe atrophy and was unable to avoid reaching to the place she was fixating (Carey, 2000).

Studies of the constant errors also lead to the conclusion that there is a common neural representation of intended eye and hand movements to the same target. For example, spatial errors of horizontal saccades of human subjects were larger for targets 20° away than for targets 10^*o*^ away and they were larger for briefly presented targets than for persistent targets (Fisk and Goodale, 1985). The same was true of their reaching movements so that the correlation between the end point of eyes and forelimb was statistically significant (*t* = 3.3, *p* < 0.01) albeit low (*r* = 0.17). In another study, Kattoulas et al. (2008) focused on the constant errors of saccades toward memorized targets and the reaching movements they accompany. The endpoints of saccades executed by monkeys toward the memorized location of visual targets land above the targets, displaying an upward shift irrespective of target location (Gnadt et al., 1991; Stanford and Sparks, 1994; White et al., 1994). Arm-pointing movements, on the other hand, display a very different pattern of constant errors. Subjects tend to direct their movements away from cardinal and toward oblique directions (Smyrnis et al., 2000, 2007). Given these two very different biases, it is reasonable to ask what pattern of errors would materialize when subjects are asked to both look and point toward memorized targets. To address this question, adult rhesus monkeys were trained to both reach and look toward targets in a center out memory task. Systematic saccade error was in this case found to co-vary with reaching error (Kattoulas et al., 2008). The percentage of the variance of saccadic errors accounted by reaching errors could be as high as 34% (*p* < 10^−5^). It might also be more meaningful to study the early part of the eye and pointing movements which rely on the initial displacement command rather than later on-line corrections operating for the hand. This is what Frens and Erkelens (1991) did in a study that had subjects quickly fixate and point at unexpectedly appearing eccentric targets. When a gap was introduced between extinction of the fixation point and target presentation, the error rate in the initial movement direction of saccade and hand movements increased to about 50%. Yet, saccade and hand movements were always made in the same direction, again suggesting that eye and hand can be guided by the same displacement command.

To summarize, the bulk of presently available evidence indicates that there is a modest but often highly significant correlation between the reaction times of saccades and hand movements of humans and monkeys executing coordinated eye-hand movements to the same target. The present report also demonstrates that the premotor cortex contains Meq cells discharging for movements of both the eyes and the hand and that the onset of their bursts is coupled to the onset of saccades or the onset of the hand movement and often of both effectors.

### Possible role of meq neurons

It has been argued that rather than drive eye movements, saccadic responses in the premotor cortex provide spatial information to their targets (Pesaran et al., 2010). In a similar vein the saccadic activity of reach related neurons of the parietal reach region (PRR) is thought not to reflect saccade planning or execution (Snyder, 2000). We feel that Meq cells may be more directly involved in the control of eye and hand movements albeit encoding somewhat more abstract variables, such as an effector independent desired displacement vector anchored on the line of sight. The possibility that premotor cortex neurons encode an abstract retinotopic displacement vector (such as the intended displacement of a cursor on a screen) rather than desired limb trajectory or the muscle activations that would bring this about was shown by Shen and Alexander (1997). These authors trained monkeys to move a cursor on a screen with the help of a handheld joystick and manipulated the spatial mappings between joystick and cursor. In the “non-rotated” case forward and rightward movements of the joystick moved the cursor upward and rightward, respectively, while rightward and backward movements of the joystick in the “rotated” case moved the cursor upward and rightward, respectively. Of the directionally tuned PMd cells with premovement-related activity they encountered there were three times as many that discharged for the intended displacement of the cursor (34/66 = 51%) as compared to those that discharged for limb trajectory (9/66 = 14%). Similarly, Kuang et al. (2016) used reversing prisms to dissociate visual from physical movement goals and found that posterior parietal neurons could encode either.

**Figure 15:**
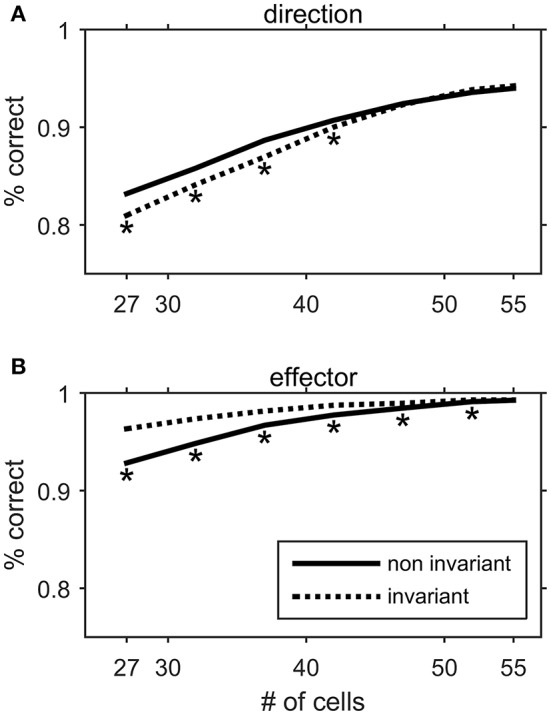
**Performance of an artificial neural network in decoding the direction (A)** or the effector **(B)** of a movement from the movement related discharges of Meq neurons. The input layer consisted of a varying mixture of units with effector invariant movement fields and units with effector non-invariant movement fields. The solid line represents the performance of the network when a progressively larger number of non-invariant Meq units are eliminated from the network's input. The dashed line represents the performance when invariant Meq units are eliminated from the input. Asterisks denote statistically significant difference in performance between populations of the same size but different composition (*t*-test, *p* < 0.05).

The existence of non-invariant Meq cells may seem at odds with the notion that these neurons encode an abstract version of the intended displacement vector not anchored to the effector employed. It is tempting to speculate that cells with “non-invariant” movement fields complement the neurons with “effector invariant” fields in that both may be needed to optimize the choice of effector and the representation of movement metrics. To address this issue, we trained a three layer artificial neural network (ANN) to infer the direction of the movement (to one of eight targets) and the effector executing it (eye, hand or eye/hand) from the average intensity of discharge of Meq cells. The decoding ANN we used is described in the Methods. It reached a high percentage of correct inferences (close to 100% for the effector and more than 90% for the movement direction) when the whole population of our Meq cells (*N* = 55) was used (Figure 15). To examine the potential contribution of Meq cells with “invariant” movement fields we eliminated a progressively larger number of them from the input layer. We then studied the percentage of correct responses it provided as it attempted to infer the direction of the movement (Figure 15A, dashed) and the identity of the effector (Figure 15B, dashed). Similarly, to examine the potential contribution of Meq cells with “non-invariant” fields we progressively eliminated such neurons from the input of the ANN and examined the percentage of correct responses regarding the direction of the movement (Figure 15A, solid) and the identity of the effector (Figure 15B, solid). Elimination was random and was repeated 200 times for each number (n_i_) of neurons eliminated. Each one of the 200 resulting ANNs was retrained and their performance was averaged to obtain a value characteristic of networks reduced by n_i_ neurons. These values are plotted in Figure 15 as a function of 55-n_i_. The two curves (solid and dashed) remained identical to each other and changed little when the first 7 neurons were eliminated, but diverged when a larger number of cells was removed. Progressive elimination of Meq cells with “invariant” fields led to a significant drop in the number of correct inferences concerning the direction of the movement (Figure 15A, dashed). On the other hand, progressive elimination of Meq cells with “non-invariant” movement fields led to a significant drop in the number of correct inferences concerning the effector to be used (Figure 15B, solid). Thus, it would seem that both subpopulations of Meq neurons are needed to minimize errors when programming movements of different effectors to different targets.

The notion that Meq cells send the same signal to both eye and hand movers does not imply that the transformations needed to execute hand and eye movements are simple. In fact the opposite is likely to prove true. Firstly, sending the same vector of desired displacement, $\vec{AB}$, to both eyes and hand suffices only when both effectors start from the same point, A. When the hand movement starts from a different point (C), to obtain the correct vector of desired hand displacement ($\vec{CB}$) to the same target, B, one further needs to subtract the vector $\vec{AC}$ (i.e., the vector of oculocentric initial hand position) from $\vec{AB}$. This scheme is consistent with the notion that the brain uses an oculocentric frame of reference to encode arm movements (Batista et al., 1999; Medendorp et al., 2005). It is also consistent with the use of vector subtraction (Moschovakis et al., 1988, 1996; Moschovakis, 1996) rather than position comparators to compute desired displacement signals. Assuming that it is amplitude control rather than position control (Bock and Eckmiller, 1986; Ghez et al., 1995; Rossetti et al., 1995) that the brain implements in eye-hand coordination, several processing steps are needed to transform the signal carried by Meq cells into that present in motoneurons. (1) Gating/decision filters allowing decoupling of eye movement commands from hand movement commands and thus of eye movements from hand movements. (2) Vector decomposition and spatiotemporal transformation to change the labeled line code employed by sensory systems and higher levels of sensorimotor interfaces to the frequency code usually employed by motor systems. (3) Inverse kinematic transformation to obtain the desired changes of joint angles from the desired endpoint displacements. (4) Inverse dynamic transformation to match the frequency content of the signals to the impedance of the effectors (these can differ a lot as in the case of eyes and hands). Processes 2–4 are likely to take place within subcortical structures and some of the mechanisms underlying them have attracted considerable interest in particular as concerns saccades (e.g., Robinson, 1973; Scudder, 1988; Moschovakis, 1994; Dean, 1995, 1996; Bozis and Moschovakis, 1998; Moschovakis et al., 1998b; Sklavos and Moschovakis, 2002; Kardamakis et al., 2010; Joshua et al., 2013). It is worth exploring if gating/decision processes that decouple eye movements from hand movements take place within the premotor cortex.

## Author contributions

EN and AM designed and performed the experiments analyzed the data and wrote the manuscript.

### Conflict of interest statement

The authors declare that the research was conducted in the absence of any commercial or financial relationships that could be construed as a potential conflict of interest.
